# An Awareness of Pharmacovigilance Among Healthcare Professionals Due to an Underreporting of Adverse Drug Reactions Issue: A Systematic Review of the Current State, Obstacles, and Strategy

**DOI:** 10.2174/0115748863276456231016062628

**Published:** 2023-10-16

**Authors:** Risani Andalasia Putri, Zullies Ikawati, Fita Rahmawati, Nanang Munif Yasin

**Affiliations:** 1 Department of Pharmacy, Dharmais National Cancer Hospital, RS, Kanker Dharmais, Jl. S. Parman Kav, 84 - 86, West Jakarta, Indonesia;; 2 Department of Pharmacology and Clinical Pharmacy, Faculty of Pharmacy, Universitas Gadjah Mada, Sekip Utara Street, Yogyakarta, Indonesia;; 3 Department of Pharmacy, Universitas Gadjah Mada, Bulaksumur, Caturtunggal, Kec. Depok, Kabupaten Sleman, Daerah Istimewa Yogyakarta, 55281, Indonesia

**Keywords:** ADR, pharmacovigilance, HCP, underreporting factors, KAAP, LOS

## Abstract

**Background:**

Healthcare professionals play an essential role in reporting adverse drug reactions as part of pharmacovigilance activities. However, adverse drug reactions reported by healthcare professionals remain low.

**Objective:**

The aim of this systematic review was to investigate healthcare professionals' knowledge, awareness, attitude, and practice on pharmacovigilance and adverse drug reaction reporting, explore the causes of the underreporting issue, and provide improvement strategies.

**Methods:**

This systematic review was conducted using four electronic databases for original papers, including PubMed, Scopus, Google Scholar, and Scholar ID. Recent publications from 1^st^ January 2012 to 31^st^ December 2022 were selected. The following terms were used in the search: “awareness”, “knowledge”, “adverse drug reaction”, “pharmacovigilance”, “healthcare professional”, and “under-reporting factor”. Articles were chosen, extracted, and reviewed by the two authors.

**Results:**

Twenty-five studies were selected for systematic review. This review found that 24.8%–73.33% of healthcare professionals were unaware of the National Pharmacovigilance Center. Around 20%–95.7% of healthcare professionals have a positive attitude toward pharmacovigilance and adverse drug reaction reporting, while 12%–60.8% of healthcare professionals have experience reporting any adverse drug reaction in their practice. The most frequently highlighted barriers to pharmacovigilance were a lack of awareness and knowledge regarding what, when, and to whom to report.

**Conclusion:**

Underreporting issues require immediate attention among healthcare professionals due to a lack of awareness and knowledge of pharmacovigilance and adverse drug reaction reporting. Educational and training program interventions have been suggested by most studies to address these issues.

## 1 INTRODUCTION

Drug use is always faced with the risk of adverse drug reaction (ADR) [1], ranging from mild reactions to life-threatening events [2–4]. ADR is defined as a response to a drug that is noxious and unwanted, occurring at doses normally used in humans for prophylaxis, diagnosis, treatment of disease, or the modification of physiological function [5]. ADRs can have significant economic implications due to their potential to lead to heightened rates of hospitalization, morbidity, mortality, and associated costs [6-10]. The incidence of ADR in hospitalized patients is estimated to occur in 6.5% of patients, 0.32% of whom are potentially fatal, and 28% of these are preventable [11, 12]. The data showed that the mortality rate and length of stay (LOS) of patients with ADR were higher (19.18% and 8.25%) than those without ADR, with medical costs for patients experiencing ADR increasing by 19.86% on average [11]. However, the importance of the problem may actually be underestimated since, in many cases, ADRs are not suspected, which leads to under-reporting [13, 14].

The detection and reporting of ADRs are highly important in order to ensure the safety of drug use for patients and the public's health. Due to the increasing number of drugs on the market and insufficient safety data from premarketing phase trials, drug safety must be monitored on a regular basis [5, 11, 12]. The activities associated with the detection, assessment, understanding, and prevention of ADRs are known as pharmacovigilance (PV), whose main goal is to minimize the harm caused by medications by rationalizing their use [5].

Spontaneous reporting is the most frequently used strategy in PV and the most effective method for generating signals on new or uncommon ADRs [13–17]. This approach has the capability to offer a substantial amount of data at minimal expense [18-21]. It encourages the rapid detection of potential alarm signals associated with medication use through the early detection of new ADRs [22]. However, under-reporting is a serious flaw in the PV system, and it occurs for a variety of reasons, including lack of awareness about the ADR reporting system [23, 24], lack of time [25, 26], difficulty in ADR decision-making [27], lack of information provided by patients [28].

Healthcare professionals [HCPs] are the most strategic key players in the supervision of drug safety as the first person to deal directly with the ADRs that were experienced by patients during practice [29, 30]. Adequate knowledge and skills about the safe use of drugs in clinical practice are important, but most HCPs have limited PV competencies, thereby needing some interventions to enhance pharmacovigilance activities [16, 31, 32]. The purpose of this systematic review is to assess the current state of knowledge, awareness, attitude, and practice (KAAP) regarding PV and ADR reporting among HCPs, to identify current barriers to ADR reporting, and to suggest improvements. This can assist in determining the current need for PV and ADR reporting.

## 2 MATERIALS AND METHODS

This systematic review was carried out in compliance with the Preferred Reporting Items for Systematic Reviews and Meta-analyses (PRISMA) statement [33]. Recent publications from 1^st^ January 2012 to 31^st^ December 2022 were selected because they were considered to represent the current state of awareness and knowledge in the countries where the studies were conducted.

### 2.1 Search Strategy

We searched four electronic databases for original papers, including PubMed, Scopus, Google Scholar, and Scholar ID. The search was commenced by applying the terms “awareness”, “knowledge”, “adverse drug reaction”, “pharmacovigilance”, “healthcare professional”, and “under-reporting factor”. Search terms were combined using the operators “AND” and “OR” in order to ensure that all relevant articles were located. Only accessible full-text articles from observational studies conducted between 1^st^ January 2012 and 31^st^ December 2022 were included, focusing on HCP awareness and knowledge of ADR and PV, factors contributing to underreporting, and suggestions for improvement. Interventional studies on ADR reporting, articles without complete text accessibility, and review articles were excluded.

### 2.2 Data Screening

After removing all duplicate titles, we screened abstracts and, subsequently, full texts against the inclusion criteria. Both authors assessed eligible studies independently. A preference on which studies to include in the review was discussed to achieve agreement. The flowchart of the study that was retrieved is depicted in Fig. (**1**).

### 2.3 Data Extraction, Synthesis, and Quality Assessment

The data were extracted by the lead author and confirmed independently by the co-author. We also checked the list of references manually to identify potentially eligible studies. For each study, data were extracted related to author, publication year, country, study design, response rate and sample size, instrument used, outcome measure, and results. The extracted data were not combined statistically because of the differences found in the study designs, study instruments, and participants recruited. The data were placed into Microsoft Excel and analyzed thematically to aid comparison between studies.

The quality assessment of studies was conducted using the Joanna Briggs Institute Critical Appraisal Checklist Tools (https://jbi.global/critical-appraisal-tools) for analyti-cal cross-sectional studies. Each checklist criterion was rated as ‘yes’, ‘no’, ‘unclear’, or ‘not applicable’. The authors independently assessed the quality of the study, discussed it, and resolved any discrepancies (Appendix **A**).

## 3 RESULT

**Fig. (1):**
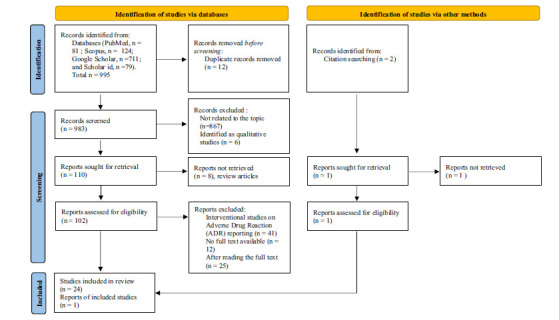
PRISMA diagram of retrieved article.

A total of 995 studies were retrieved from the four scientific databases (PubMed, Scopus, Google Scholar, and Scholar ID) for analysis. After screening titles and abstracts for duplicate and irrelevant studies, 983 papers were excluded. Review articles with no full text available and the studies that did not meet the inclusion criteria were also excluded. Eligible studies from the reference list were included in this review. Finally, 25 studies were selected for the systematic review (Fig. **1**).

### 3.1 Study Characteristics

All 25 studies included in the systematic review were cross-sectional observational surveys using either manual or online questionnaires conducted among HCPs and published between 1^st^ January 2012 and 31^st^ December 2022. The studies were conducted in Saudi Arabia, India, South Africa, Nepal, Pakistan, Bosnia and Herzegovina, Southwestern Nigeria, Vietnam, Jordan, Turkey, Ghana, and Indonesia. HCPs were surveyed in the studies, with sample sizes ranging from 33 [34] to 2091 [35]. The critical appraisal results of the quality of each study included are described in Appendix **A**. The majority of studies included did not have data related to identification and strategies to deal with confounding factors. The other three studies could have used more appropriate statistical analysis. A study did not clearly inform the validation process. All of the 25 studies obtained more than 50% “yes” in the checklist, met the quality criteria, and were selected for systematic review.

### 3.2 Pharmacovigilance Knowledge

Knowledge is defined as a theoretical or practical understanding of a subject [36]. In this review, knowledge-related items included a fundamental understanding of the terms ADR and PV, the purpose of reporting, and who, what, and where to report an ADR. Knowledge of PV and ADR reporting was primarily assessed using multiple-choice response options ranging from 6 to 16 questions [24, 30, 35, 37-43]. The correct responses were determined using the WHO definition [44].

Most of the studies included in this systematic review reported insufficient knowledge of PV and ADR reporting, with reported rates ranging from 15% to 65.8%. Two of twenty-five studies demonstrated adequate knowledge of HCPs (Table **1**) [45, 46]. Only a few studies have been conducted to compare the level of knowledge among HCPs. Three studies compared the knowledge of medical doctors, nurses, and pharmacists and found that pharmacists have a significantly greater understanding of PV and ADR reporting (*p <*0.05) [37, 46-48]. Other studies found doctors possess a higher level of knowledge than other professions (*p* <0.05) [40] and nurses have a greater understanding of PV and ADR reporting (*p* <0.001) [42, 49]. In 2016, Gurmesa & Dedefo reported that doctors and pharmacists have better knowledge of PV and ADR reporting than nurses (89.18%) [38].

### 3.3 Pharmacovigilance Awareness

Awareness was the main outcome of four studies in this review [40, 48, 50, 51]. However, the level of awareness was also reported in other studies. Out of 10, 8 studies on PV and ADR reporting awareness indicated a low level of awareness among HCPs [24, 25, 30, 34, 40, 43, 50, 52]. Only two studies reported good awareness among HCPs on PV and ADR reporting (Table **1**) [35, 49]. This study revealed that a considerable proportion of HCPs exhibited a lack of awareness regarding the existence of the national PV center, with percentages ranging from 24.8% to 73.33%. Some studies compared the levels of awareness among HCPs, and the findings suggest that the pharmacist had the highest percentage of PV awareness, ranging from 57.14% to 88.9% [26, 34, 43]. Nurses and pharmacists were slightly more aware of the purpose of PV than physicians (*p*=0.01) [39]. The study conducted by Alshammari (2015) reported that awareness of PV and ADR reporting was better among pharmacists and medical doctors than among nurses [37]. Meanwhile, another study conducted demonstrated doctors have more awareness about PV and ADR reporting compared to other HCPs (*p*<0.05) [52].

### 3.4 Pharmacovigilance Attitude

Attitudes toward PV and ADR reporting were assessed using multiple-choice response options ranging from 5 to 17 questions [25, 26, 30, 34, 38, 39, 41-43, 50] and using a 5-point Likert [35, 37, 49]. Additionally, it was noted that certain questions were used to assess perception rather than attitude [37, 40, 53]. The most frequently asked question is concerned with the agreement on the critical nature of ADR reporting and the fact that it is an obligation for HCPs. The included studies found that between 20% and 95.7% of the participants had a positive attitude towards PV and ADR reporting. Out of 25, 19 studies assessed attitude, and three studies focused on perception rather than attitude. Eleven studies found that HCPs generally have a positive attitude toward PV [24, 25, 35, 39, 45-48, 50, 51, 54], while one study demonstrated a moderately positive attitude [49] and four studies found that HCPs have a low attitude toward PV (Table **1**) [53]. Only one study reported that pharmacists [89.5%] have a good attitude toward ADR reporting when compared to other HCPs [38].

### 3.5 ADR Reporting Practices

This review captures the ADR that has been reported by HCPs. Only fourteen studies reported completely on both HCPs' experience witnessing and reporting ADR, ranging from 12% to 97.9% and from 12% to 61% for experience witnessing and reporting, respectively (Appendix **B**) [24, 25, 30, 34, 35, 38, 39, 42, 43, 45, 47, 51, 53, 55]. Two studies reported that HCPs have witnessed ADR, which ranges from 34.4% to 98.8% [48, 49]. Two other studies reported that HCPs had documented ADR in the patient follow-up chart but had never reported ADR in their practices [43, 55]. Meanwhile, one study demonstrated that 11.7% of HCPs did not report to the proper place, and only 9.1% reported ADR to the Ministry of Health [54].

### 3.6 Factors that Discourage ADR Reporting

There are numerous reasons for not reporting ADR, which may contribute to the issue's underreporting among HCPs. This review summarizes the study's main findings on the level of KAAP, discouraging factors, and suggestions for ADR reporting among HCPs (Table **1**). Six major reasons were identified in this review: lack of awareness and knowledge on what, when, and to whom to report ADR; lack of time; unavailability of the reporting form; uncertainty regarding the suspected drug; workload on taking care of patients; and no incentive or remuneration (Table **2**). Some studies identified discouraging factors, such as the lack of information provided by patients [40], the disclaiming of responsibility for ADR reporting [53], the complexity of the reporting system [35], the lack of feedback from concerned bodies [43], the lack of PV training [25], legal liability concerns [54], and the lack of widespread promotion by relevant authorities [37].

### 3.7 Strategies to Improve ADR Reporting

We summarize the suggestions from each study for improving ADR reporting Table **1**. Most of the studies reviewed [19 studies] emphasize the importance of education and training in improving KAAP for PV and ADR reporting [24, 26, 34, 35, 37–39, 42, 43, 47-50, 52-57]. Other recommendations are the inclusion of PV in the undergraduate curriculum of health care education [30, 45, 47, 50], simplifying the ADR reporting [35, 40, 43]; providing feedback from reporting center, reminders, and advertisement [24], implementing strong regulations for ADR reporting and giving some financial incentives to HCPs [35], collaboration between regulatory bodies, the Ministry of Health, HCPs, academia, and pharmaceutical industries [41], building up a close relationship between PV center and HCPs [40], and developing training modules and making report formats available and easy to access to improve ADR reporting [38, 51] which might also be recommended by the studies included to improve ADR reporting.

## 4 DISCUSSION

The tragedy of thalidomide, which led to the development of PV, demonstrates that drug safety cannot be omitted [1]. HCPs play a pivotal role in the successful implementation of PV activities. Nevertheless, under-reporting of ADR among HCPs remains an issue that must be remedied promptly using a particular strategic approach [58, 59]. This review evaluates KAAP on HCPs in order to provide an overview of the primary reasons and propose strategic solutions. The studies that evaluated the KAAP of PV and ADR reporting by HCPs were included in this review. Most of the published studies evaluated KAAP among each professional separately, while only a few focused on HCPs in the same health facility. Our review highlights the level of KAAP of PV and ADR reporting among HCPs, mainly the reasons for not reporting any ADR by HCPs. It summarizes proposed strategies to improve ADR reporting by HCPs.

A validated questionnaire was used to assess KAAP on PV and ADR reporting among HCPs. The most widely used questionnaire was the KAP questionnaire, with variations in the number of questions for each domain to be measured (domain knowledge, awareness, attitude, and practice). For the knowledge domain, the questions related to the PV and ADR definitions and the reporting system. Meanwhile, for the attitude domain, the most frequently asked question is concerned with the agreement on the critical nature of ADR reporting and the fact that it is an obligation for HCPs. The level of practice was obtained from the percentage of HCPs' ADR experiences and ADR reporting. The lack of a standardized, validated assessment tool and the variety of questionnaire items pose significant obstacles to the study of this topic area [60]. Variations in the criteria for determining the cut-off point and characteristics in the study settings may lead to a disparity in ADR knowledge among study participants [27, 61]. The presence of a gold standard should be taken into account while evaluating various dimensions of knowledge related to PV among HCPs [20]. This facilitates a comprehensive evaluation that is valuable for identifying appropriate strategies that focus on enhancing KAAP among HCPs involved in PV activity.

The majority of the studies included in this review indicated a lack of knowledge of PV and ADR reporting. Most studies also indicated a low level of awareness regarding PV and ADR reporting by HCPs [24, 25, 30, 34, 40, 43, 50, 52]. The terms “knowledge” and “awareness” were used interchangeably to refer to the theoretical or practical understanding of ADR reporting in relation to the ADR and PV concepts [61]. In this review, we also found that the terms “knowledge” and “awareness” have a similar meaning, as shown by the outcome being measured. There are a limited number of HCPs with knowledge and awareness of PV around the world [27, 62-65]. In this review, several studies revealed that the HCPs were familiar with the terms “PV” and “ADR”, but most of them were still unaware of the existence of a PV center. This review highlights that a number of studies have indicated that HCPs are familiar with the terms “PV” and “ADR”. However, it is noteworthy that a significant proportion of HCPs remain unaware of the existence of PV centers. This phenomenon has been confirmed by previous studies carried out in various countries. They have also identified a lack of awareness among HCPs regarding the existence of international, national, and hospital-based PV centers [60, 63].

It might be argued that knowledge and awareness have certain characteristics in common, thereby emphasizing the significance of education as an approach to providing HCPs with access to pertinent information. The level of HCP knowledge regarding PV is influenced by how much HCPs were exposed to PV information and education, whether obtained from formal education (university-based) or through PV training [19, 20, 29, 66, 67]. Limited financial resources are the main factor behind the low level of HCP knowledge regarding PV in low- and middle-income countries [68, 69]. Consequentially, a limited number of professionals have a good attitude and practice regarding PV and ADR reporting.

Most HCPs from the included studies showed a positive attitude toward PV and ADR reporting [24, 25, 35, 39, 45-48, 50, 51, 54]. Furthermore, it was clearly observed that about one-fourth of HCPs were not interested in reporting suspected ADR [27]. The majority of HCPs agree that reporting ADRs is their obligation. However, it is noteworthy that some HCPs exhibit a lack of enthusiasm when it comes to reporting ADRs encountered. Previous studies have similarly revealed a consistent finding [63, 70, 71]. HCPs' positive attitudes regarding ADR reporting are crucial since understanding these attitudes is important for developing the right strategies to increase HCPs' engagement in ADR reporting [56, 72].

This review captures the ADR that has been reported by HCPs, ranging from 12% to 60.8%. Although HCPs play an important role in PV activity, the rate of ADR reporting by HCPs is low in this study. This finding was found in many studies, with a range between 12% and 40.5% [31, 73, 74]. Several questions used to assess PV and ADR reporting practices, such as “Have you ever witnessed or reported ADRs?”, relied on respondents' memories. This could lead to response biases. Surprisingly, we found in two studies that HCPs had never reported any ADR in their routine practice. Still, they documented ADR in the patient follow-up chart, with the main reason being the unavailability of the reporting form [43, 55]. In this current digital era, the unavailability of forms should no longer be an excuse. PV centers in many countries have developed electronic reporting through both web-based and mobile applications [75-79]. Assessing the necessity for comprehensive data on ADRs that are integrated into electronic medical records [EMRs], particularly for hospitalized patients, is of the utmost importance [77]. This is due to the fact that hospitals exhibit the highest rate of ADR reporting compared to other healthcare institutions or communities [70, 80-82]. This measure is anticipated to enhance both the quantity and quality of ADR reporting [83].

Current study findings indicate that pharmacists show greater levels of awareness, knowledge, attitude, and practice in comparison to other HCPs. A consistent finding was also reported in previous studies [56, 59, 64, 70, 82]. The fact that a higher percentage of pharmacists than other HCPs are aware of and have good knowledge, attitude, and practice of the ADR and PV concept systems may be related to pharmacists’ education about drug safety and the fact that ADR report forms are required to be channeled to and through the pharmacy [71, 84].

We also summarized the main reasons for HCPs failing to report any ADR. There are six primary reasons documented in this review, including the lack of awareness and knowledge on what, when, and to whom to report ADR; the lack of time; the unavailability of the ADR reporting form; uncertainty regarding suspected drug; overload of work on patient management; and no incentive or remuneration. Similar findings were also reported in previous studies [62, 80, 85-87]. HCPs may occasionally refrain from reporting ADRs due to the inherent difficulty of definitively establishing a causal relationship between a drug and an ADR [22, 88, 89]. Nevertheless, adhering to the fundamental principle of PV, it is advisable to report any suspicion, as this can help raise awareness and contribute to safeguarding public health. A study conducted by Ali *et al*. [64] revealed a noteworthy finding: HCPs encountered a barrier in reporting ADR due to the absence of access to a competent professional environment for discussing such incidents. Interprofessional collaboration may be regarded as a potential answer to this issue. These variables can at least provide a framework for determining how to address the problem of underreporting.

Our review also outlines each study's recommendations regarding improving ADR reporting, as most of the studies identified the main issue as HCPs' lack of awareness and knowledge of PV and ADR reporting. Most studies in this review revealed educational and training interventions as a priority to address this issue. HCPs’ knowledge of PV and ADR reporting is important since they are still students [90–93]. A few universities offer PV programs or education on all PV-related topics. Therefore, it is necessary to review the need for PV education in HCPs' student curricula [32, 91, 92]. PV competencies were also affected by healthcare school types, academic level, and previous training [91]. Gap analysis and needs assessment should guide education and training methods [20, 29, 66, 67, 94-96]. Furthermore, the findings of this review demonstrate the wide range of KAAP among HCPs. This variation can be attributed primarily to differences in exposure to PV education and training programs [93]. HCPs must be knowledgeable and competent to report ADRs [90, 97]. Our findings are consistent with those of a previously published review, which examines the PV competencies of each HCPs [27, 60, 61]. As a result, there are a limited number of specialists capable of conducting medication safety assessments and enhancing risk management [20]. The past decade has witnessed significant developments in digital technology, which have therefore resulted in a heightened utilization of electronic reporting systems with the aim of enhancing ADR reporting. There is a need for more rigorous research to examine the effects of various electronic approaches in order to comprehensively assess their potential for enhancing ADR reporting [98]. Therefore, specific strategies should be designed in order to improve KAAP of healthcare professionals to address ADR underreporting-related issues. The majority of the studies included in this review indicated a lack of knowledge of PV and ADR reporting. Most studies also indicated a low level of awareness regarding PV and ADR reporting by HCPs [24, 25, 30, 34, 40, 43, 50, 52]. The terms “knowledge” and “awareness” were used interchangeably to refer to the theoretical or practical understanding of ADR reporting in relation to the ADR and PV concepts [61]. In this review, we also found that the terms “knowledge” and “awareness” have a similar meaning, as shown by the outcome being measured. There are a limited number of HCPs with knowledge and awareness of PV around the world [27, 62-65]. In this review, several studies revealed that the HCPs were familiar with the terms “PV” and “ADR”, but most of them were still unaware of the existence of a PV center. This review highlights that a number of studies have indicated that HCPs are familiar with the terms “PV” and “ADR”. However, it is noteworthy that a significant proportion of HCPs remain unaware of the existence of PV centers. This phenomenon has been confirmed by previous studies carried out in various countries. They have also identified a lack of awareness among HCPs regarding the existence of international, national, and hospital-based PV centers [60, 63].

It might be argued that knowledge and awareness have certain characteristics in common, thereby emphasizing the significance of education as an approach to providing HCPs with access to pertinent information. The level of HCP knowledge regarding PV is influenced by how much HCPs were exposed to PV information and education, whether obtained from formal education [university-based] or through PV training [19, 20, 29, 66, 67]. Limited financial resources are the main factor behind the low level of HCP knowledge regarding PV in low- and middle-income countries [68, 69]. Consequentially, a limited number of professionals have a good attitude and practice regarding PV and ADR reporting.

Most HCPs from the included studies showed a positive attitude toward PV and ADR reporting [24, 25, 35, 39, 45-48, 50, 51, 54]. Furthermore, it was clearly observed that about one-fourth of HCPs were not interested in reporting suspected ADR [27]. The majority of HCPs agree that reporting ADRs is their obligation. However, it is noteworthy that some HCPs exhibit a lack of enthusiasm when it comes to reporting ADRs encountered. Previous studies have similarly revealed a consistent finding [63, 70, 71]. HCPs' positive attitudes regarding ADR reporting are crucial since understanding these attitudes is important for developing the right strategies to increase HCPs' engagement in ADR reporting [56, 72].

This review captures the ADR that has been reported by HCPs, ranging from 12% to 60.8%. Although HCPs play an important role in PV activity, the rate of ADR reporting by HCPs is low in this study. This finding was found in many studies, with a range between 12% and 40.5% [31, 73, 74]. Several questions used to assess PV and ADR reporting practices, such as “Have you ever witnessed or reported ADRs?”, relied on respondents' memories. This could lead to response biases. Surprisingly, we found in two studies that HCPs had never reported any ADR in their routine practice. Still, they documented ADR in the patient follow-up chart, with the main reason being the unavailability of the reporting form [43, 55]. In this current digital era, the unavailability of forms should no longer be an excuse. PV centers in many countries have developed electronic reporting through both web-based and mobile applications [75–79]. Assessing the necessity for comprehensive data on ADRs that are integrated into electronic medical records (EMRs), particularly for hospitalized patients, is of the utmost importance [77]. This is due to the fact that hospitals exhibit the highest rate of ADR reporting compared to other healthcare institutions or communities [70, 80-82]. This measure is anticipated to enhance both the quantity and quality of ADR reporting [83].

Current study findings indicate that pharmacists show greater levels of awareness, knowledge, attitude, and practice in comparison to other HCPs. A consistent finding was also reported in previous studies [56, 59, 64, 70, 82]. The fact that a higher percentage of pharmacists than other HCPs are aware of and have good knowledge, attitude, and practice of the ADR and PV concept systems may be related to pharmacists’ education about drug safety and the fact that ADR report forms are required to be channeled to and through the pharmacy [71, 84].

We also summarized the main reasons for HCPs failing to report any ADR. There are six primary reasons documented in this review, including the lack of awareness and knowledge on what, when, and to whom to report ADR; the lack of time; the unavailability of the ADR reporting form; uncertainty regarding suspected drug; overload of work on patient management; and no incentive or remuneration. Similar findings were also reported in previous studies [62, 80, 85-87]. HCPs may occasionally refrain from reporting ADRs due to the inherent difficulty of definitively establishing a causal relationship between a drug and an ADR [22, 88, 89]. Nevertheless, adhering to the fundamental principle of PV, it is advisable to report any suspicion, as this can help raise awareness and contribute to safeguarding public health. A study conducted by Ali *et al*. [64] revealed a noteworthy finding: HCPs encountered a barrier in reporting ADR due to the absence of access to a competent professional environment for discussing such incidents. Interprofessional collaboration may be regarded as a potential answer to this issue. These variables can at least provide a framework for determining how to address the problem of underreporting.

Our review also outlines each study's recommendations regarding improving ADR reporting, as most of the studies identified the main issue as HCPs' lack of awareness and knowledge of PV and ADR reporting. Most studies in this review revealed educational and training interventions as a priority to address this issue. HCPs’ knowledge of PV and ADR reporting is important since they are still students [90-93]. A few universities offer PV programs or education on all PV-related topics. Therefore, it is necessary to review the need for PV education in HCPs' student curricula [32, 91, 92]. PV competencies were also affected by healthcare school types, academic level, and previous training [91]. Gap analysis and needs assessment should guide education and training methods [20, 29, 66, 67, 94-96]. Furthermore, the findings of this review demonstrate the wide range of KAAP among HCPs. This variation can be attributed primarily to differences in exposure to PV education and training programs [93]. HCPs must be knowledgeable and competent to report ADRs [90, 97]. Our findings are consistent with those of a previously published review, which examines the PV competencies of each HCPs [27, 60, 61]. As a result, there are a limited number of specialists capable of conducting medication safety assessments and enhancing risk management [20]. The past decade has witnessed significant developments in digital technology, which have therefore resulted in a heightened utilization of electronic reporting systems with the aim of enhancing ADR reporting. There is a need for more rigorous research to examine the effects of various electronic approaches in order to comprehensively assess their potential for enhancing ADR reporting [98]. Therefore, specific strategies should be designed in order to improve KAAP of healthcare professionals to address ADR underreporting-related issues.

## CONCLUSION

Our study finds that a lack of awareness and knowledge of PV and ADR among HCPs is the main cause of underreporting issues. Education and training initiatives to improve knowledge and awareness were the most recommended interventions. However, the knowledge, awareness, attitude, and practice levels obtained among HCPs can be used to guide the formulation of appropriate educational strategies at either the hospital or community setting or in a specific region.

## Figures and Tables

**Table 1:** Summary of the results of articles.

**S. No.**	**Author (Years)**	**Country**	**Study Design**	**Setting**	**Focusing Group**	**Response Rate & Sample Size**	**Instrument**	**Outcomes Measures (Level of KAAP)**				**Main Reasons for Not Reporting ADR**	**Suggestion**
								**Knowledge**	**Awareness**	**Attitude**	**Practice**		
1	Apurva & Chaturvedi (2012) [50]	India	Cross-sectional	Hospital	Clinicians, pharmacists, nursing staff, and students	70.46%, (n=563)	Questionnaire for assessing Awareness, Attitude and Practice (10 questions)	N/A	Low	Good	N/A	Lack of awareness on ADR reporting system	Integration of PV into medical curriculum
2	Amrain & Bečić (2014)[57]	Bosnia and Herzegovina	Cross-sectional	Hospital and health centre	Doctors, pharmacists, dentists, technicians, and nurses	87%, (n=870)	Questionaire of knowledge, perception, practice and barrier (20 questions)	Low	N/A	Good	Low	Inadequate experience in filling out the ADR reporting forms and unavailability of ADR reporting forms	Education and training with an emphasis on the objectives of PV, completion of the ADR form, and more detail on the reporting criteria
3	Necho Mulatu (2014) [24]	Ethiopia	Cross sectional, qualitative study	Hospital	Physicians, pharmacy personnel, and nurses	100%, (n=708)	KAP Questionnaire (35 questions)	Low	low	Good	Low	Lack of familiarity on reporting system	Advertisements and reminders, face-to-face education, and reporting center feedback
4	Alshammari *et al.* (2015) [39]	Saudi Arabia	Cross-sectional	Hospital	Pharmacists, physicians, and nurses	72%, (n=322)	KAP Questionnaire (18 questions)	Low	N/A	Good	Better trend	No incentives, insufficient time, and difficulty in determining an ADR	Educational intervention and a practical training program need to be applied by the drug regulatory body
5	Gupta *et al.* (2015) [45]	India	Cross-sectional	Hospital	Doctors, nurses, and pharmacists	62.4%, (n=101)	KAP Questionnaire (20 questions)	Good	N/A	Good	Low	Lack of remuneration, lack of time, the perception that a single unreported case will not impact the ADR database, and the difficulty of determining an ADR	Inclusion of PV in the undergraduate curriculum and regular training on basic principles of PV
6	Gurmesa & Dedefo (2016) [38]	Ethiopia	Cross-sectional	Health centers and clinics	Nurses, doctors, health officers, and pharmacists	100%, (n=133)	KAP Questionnaire (20 questions)	Low	N/A	Low	Low	Lack of awareness and knowledge on what, when, and to whom to report ADR and lack of commitments of HCPs	In-House Training, direct supervision of patients by pharmacists, and making report formats available
7	Le *et al*. (2020) [35]	Vietnam	Cross-sectional	Hospital	Doctors, pharmacists, and nurses	80.4%, (n=2091)	KAP Questionnaire (35 questions)	Low	Good	Good	Low	Unavailability, complexity of reporting form and lack of time	Combination of multiple strategies, including the implementation of stringent regulations for ADR reporting, the providing of adequate training to HCPs, the simplification of the ADR reporting process using an electronic system, and the provision of financial incentives to HCPs
8	Almandil (2016) [34]	Saudi Arabia	Cross-sectional	Hospital	Physicians, pharmacists, pharmacy technicians, and nurses	82.75%, (n=33)	KAP Questionnaire (17 questions)	Low	Low	N/A	Low	Lack of awareness and knowledge of PV and ADR reporting	Establishing educational programs and strategies
9	Wangge & Akbar (2016) [41]	Indonesia	Cross-sectional	Health care	Medical doctors, nurses, and pharmacists	92%, (n=109)	KAP Qustionnaire (12 questions)	Low	N/A	Low	Low	Low level of KAP	All of its key players (regulatory bodies, the Ministry of Health, HCPs, academics, and the pharmaceutical industry) should work closely together
10	Abu Hammour *et al*. [2017] [40]	Jordan	Cross-sectional	Hospital	Medical doctors and nurses	50,7%, (n=340)	Questionnaire related to knwledge, perception and discouraging factors (27 questions)	Low	Low	Good	N/A	Lack of information provided by patients, lack of time, and do not know where and how to report	Focusing on education, developing reliable and straightforward reporting systems, and establishing close relationships between PV centers
11	Shanko & Abdela (2018) [43]	Ethiopia	Cross-sectional	Hospital	Nurses, physicians, and pharmacists	91.4%, (n=295)	KAP Questionnaire	Low	Low	N/A	Low	Unavailability of the reporting form, uncertainty of how to report, and lack of feedback from the concerned body	In-service training and an appropriate reporting system
12	Terblanche *et al*. (2017) [26]	South Africa	Cross-sectional	Hospital	Medical practitioners, nurses, pharmacists, and pharmacist assistants	24%, (n=132)	Questionnaire for knowledge, attitude and factors perceived to influence ADR reporting (19 questions)	Low	N/A	N/A	N/A	Not knowing how to report and lack of time	Appropriate training, familiarization with the PV system in hospitals, and training in its operation, as well as in-house meetings and training sessions
13	Ali *et al*. (2018) [53]	Saudi Arabia	Cross-sectional	Health centers	Doctors, nurses, pharmacists, and other health care provider	84.37%, (n=135)	KAP Questionnaire	Low	N/A	Low	Low	Disclaiming responsibility and lack of awareness	CME and training.
14	Moinuddin *et al*. (2018) [25]	Saudi Arabia	Cross-sectional	Hospital	Doctors, pharmacists, nurses, dentists, and technicians	88.6%, (n=399)	Questionnaire (Knowledge & Attitude, 29 questions)	Low	Low	N/A	N/A	Lack of PV training, workload, and legal liabilities	CME
15	Ergün *et al*. (2019) [42]	Turkey	Cross-sectional	Hospital	Doctors, nurses, and students	58%, (n=482)	KAP questionnaire (25 questions)	Low	N/A	N/A	Low	Lack of the knowledge of the national PV system	Standard, regular, and intensive education programs should be provided to pharmacologists and pharmacological contact points
16	Damodar *et al*. (2018) [52]	India	Cross-sectional study	Hospital	Doctors, nurses, and pharmacists	75.4%, (n=264)	KAP Questionnaire (10 questions for assesing Knowledge, awareness and prctice)	Low	Low	N/A	N/A	Poor knowledge towards ADRs reporting among HCP	Educational and adequate training programs
17	Nisa *et al*. (2018) [54]	Pakistan	Cross-sectional study	Hospital	Physicians and pharmacists	95.5%, (n=367)	KAP Questionnaire (27 questions)	N/A	N/A	Good	Low	Lack of knowledge regarding where and how to report ADR, lack of access to an ADR reporting form, the importance of patient care over ADR reporting, and legal liability issues	Educational training related to where and how to report ADR
18	R Adisa & Omitogun (2019) [49]	Southwestern Nigeria	Cross-sectional	Primary Healthcare Centres [PHCs]	Health extension workers, health officers, nurses, health assistants, and physicians	100%, (n=80)	KAP Questionnaire (25 questions)	low	good	good	N/A	Unavailability of the ADR reporting form and the non-existence of a formal reporting system	A need for regular mandatory education and training
19	Danekhu *et al*. (2021) [37]	Nepal	Cross-sectional	Hospital	Medical doctors, nurses, and pharmacists	100%, (n=215)	Questionnaire (Knowledge and Perception, 25 questions)	Low	N/A	Good	N/A	A lack of promotion by relevant authorities as DDA, RPC, and also the HCPs did not know where and how to report ADR	Focus on educating HCPs about ADR and how to report it
20	Sujatha *et al*. [2019] [48]	India	Cross-sectional	Hospital	Doctors, nurses, and pharmacists	71.4%, (n=250)	Questionnaire related to knowledge, awareness and practice	Low	N/A	Good	Low	Lack of knowledge	Educational and regular training programme
21	Hussain *et al*. (2021) [56]	Pakistan	Cross-sectional	Hospital	Nurses, physicians, and pharmacists	90.10%, (n=346)	KAP Questionnaire (35 questions)	Good	N/A	Good	N/A	lack of knowledge	A system of hands-on training and workshops at hospital level
22	Kumar *et al*. (2020) [30]	India	Cross-sectional	Hospital	Doctors, nurses, and pharmacists	100%, (n=100)	KAP Questionnaire	Low	Low	Low	Low	Difficulty in ADR decision-making and lack of time	Educational intervention strategies; details of PV in the undergraduate curriculum
23	Yawson *et al*. (2022) [51]	Ghana	Cross-sectional	Hospital	Doctors, nurses, and pharmacists	89%, (n=378)	Questionnaire awareness and knowledge and attitude	Low	N/A	Good	Low	Insufficient knowledge	Developing appropriate training modules
24	Aryal *et al*. (2022) [55]	Nepal	Cross-sectional	Hospital	Doctors and nurses	91.9%, (n=260)	Questionaire related to ADR reporting experience, discouraging factors and recommendation	N/A	N/A	Good	Low	Unavailability of reporting form	Training programme
25	Gordhon & Padayachee (2020) [47]	South Africa	Cross-sectional	Community service	Pharmacists, nurses, and medical doctors	87.87%, (n=297)	KAP Questionnaire (19 questions)	N/A	N/A		N/A	Patient management, lackof knowledge and attitude, and lack of time	Investigations into undergraduate PV curriculum

**Abbreviations:** HCP, Health Care Professional; ADR, Adverse Drug Reaction; PV, Pharmacovigilance; KAP, Knowledge, Attitude and Practice; KAAP, Knowledge, Awareness, Attitude, and Practice; CME, Continuing Medical Education; DDA, Departement of Drug Administration; RPC, Regional Pharmacovigilance Center; N/A, Not Available.

**Table 2:** Top six discouraging factors shown in studies.

**S. No.**	**Discouraging Factors on ADR Reporting**	**Reporting Studies**
1	Lack of awareness and knowledge on what, when and to whom to report ADR	[24, 26, 50-54, 56, 34, 38, 40-43, 47, 48]
2	Lack of time	[26, 30, 39, 40, 45, 47]
3	Unavailability reporting form	[35, 43, 49, 55, 57]
4	Uncertainty regarding the suspected drug	[30, 39, 45]
5	Workload for taking care of patients (patient management is more important)	[25, 47, 54]
6	No incentive or remuneration	[39, 45]

**Abbreviations:** ADR, Adverse Drug Reaction.

## Data Availability

All the data and supportive information are provided within the article.

## APPENDIX A. The methodological quality assessment of studies.

**Table:**

**JBI Critical Appraisal Checklist for cross-sectional studies**	**Study Number (see ** **Table 1** **)**																																															
	**1**	**2**	**3**	**4**	**5**	**6**	**7**	**8**	**9**	**10**	**11**	**12**	**13**	**14**	**15**	**16**	**17**	**18**	**19**	**20**	**21**	**22**	**23**	**24**	**25**
Were the criteria for inclusion in the sample clearly defined?	Y	Y	Y	Y	Y	Y	Y	Y	Y	Y	Y	Y	Y	Y	Y	Y	Y	Y	Y	Y	Y	Y	Y	Y	Y
Were the study subjects and the setting described in detail?	Y	Y	Y	Y	Y	Y	Y	Y	Y	Y	Y	Y	Y	Y	Y	Y	Y	Y	Y	Y	Y	Y	Y	Y	Y
Was the exposure measured in a valid and reliable way?	Y	Y	Y	Y	Y	Y	U	Y	Y	Y	Y	Y	Y	Y	Y	Y	Y	Y	Y	Y	Y	Y	Y	Y	Y
Were objective, standard criteria used for measurement of the condition?	Y	Y	Y	Y	N	Y	Y	Y	N	Y	N	Y	Y	Y	Y	Y	Y	Y	Y	Y	Y	Y	N	N	N
Were confounding factors identified?	Y	Y	NA	NA	NA	NA	Y	NA	NA	Y	NA	Y	Y	NA	Y	NA	Y	NA	NA	NA	NA	NA	NA	NA	NA
Were strategies to deal with confounding factors stated?	Y	Y	NA	NA	NA	NA	Y	NA	NA	Y	NA	Y	Y	NA	Y	NA	Y	NA	NA	NA	NA	NA	NA	NA	NA
Were the outcomes measured in a valid and reliable way?	Y	Y	Y	Y	Y	Y	Y	Y	Y	Y	Y	Y	Y	Y	Y	Y	Y	Y	Y	Y	Y	Y	Y	Y	Y
Was appropriate statistical analysis used?	Y	Y	Y	Y	N	Y	Y	Y	N	Y	N	Y	Y	Y	Y	Y	Y	Y	Y	Y	Y	Y	Y	Y	Y

**Abbreviations:** Y, Yes; N, No; U, Unclear; NA, Not Applicable.

## APPENDIX B. Experience in witnessing and reporting ADR.

**Table:**

ADR reporting Practice	Necho Mulatu (2014) [24]	Alshammari *et al.* (2015) [39]	Gurmesa & Dedefo (2016) [38]	Gupta *et al.* (2015) [45]	Le *et al.* (2020) [35]	Almandil (2016) [34]	Shanko & Abdela (2018) [43]	Terblanche *et al.* (2017) [26]	Ali *et al.* (2018) [53]	Moinuddin *et al.* (2018) [25]	Ergün *et al.* (2019) [42]	Nisa *et al.* (2018) [54]	R Adisa & Omitogun (2019) [49]	Sujatha *et al.* (2019) [48]	Kumar *et al.* (2020) [30]	Yawson *et al.* (2022) [51]	Aryal *et al.* (2022) [55]	Gordhon & Padayachee (2020) [47]
ADR experienced	16.2%	60.8%	27%	64.4%	97.9%	12%	49.2%	N/A	57.7%	71.2%	41%	N/A	98.8%	34.4%	67%	82.8%	91.9%	58.59%
ADR reported	28.5%	60.8%	38.8%	22.8%	59.3%	12%	37.3%*	12.1%	17.77%	55.1%	21%	11.7% **	N/A	N/A	61%	52.6%	45.6% *	16.50%

**Notes:** *HCPs recorded ADR in the patient follow-up chart, **HCPs didn’t report to the proper place, only 9.1% respondents report ADR to the Ministry of Health.

**Abbreviations:** ADR, Adverse Drug Reaction; HCP, Health Care Professional; N/A, Not available.

## LIST OF ABBREVIATIONS

ADR: Adverse Drug Reaction
HCPs: Healthcare Professionals
KAAP: Knowledge, Awareness, Attitude, and Practice
LOS: Length of Stay
PRISMA: Preferred Reporting Items for Systematic Reviews and Meta-analyses
PV: Pharmacovigilance

## STANDARDS OF REPORTING

PRISMA guidelines and methodology were followed.

## SUPPLEMENTARY MATERIAL

PRISMA checklist is available as supplementary material on the publisher’s website along with the published article.
